# Trauma patients and cervical spine protection in critical care: the impact of a spinal checklist on clinical care and documentation

**DOI:** 10.1186/cc11062

**Published:** 2012-03-20

**Authors:** A Chick, C Scott, H Ellis, A Tipton

**Affiliations:** 1 Sheffield Teaching Hospitals NHS Foundation Trust, Sheffield, UK

## Introduction

In October 2010 a specific online proforma for cervical spine (C-spine) assessment in the context of trauma was introduced in critical care in a large UK teaching hospital. The aim of this study is to assess the impact of the Metavision Spinal Checklist (MSC) on clinical care and documentation. Prior to October 2010, the documentation of C-spine status on admission to critical care was incomplete or unclear in over 40% of these patients.

## Methods

Patients were identified from a comprehensive critical care database. Inclusion criteria: age >16; polytrauma or traumatic brain injury; other trauma where mechanism of injury suspicious for C-spine injury; admission date after 1 October 2010, before 30 November 2011. Exclusion criteria: pre-existing spinal injury; mechanism of trauma not consistent with C-spine injury. Clinical and MSC details were recorded, including sequential forms for individual patients where the C-spine status changed (for example, C-spine cleared and hard collar removed).

## Results

A total of 62 patients met the inclusion criteria; 47% of these had been transferred from a district hospital. In patients with an MSC completed, there was 100% documentation of time, date and name of the completing critical care consultant. Seventy-five per cent of initial MSCs indicated the name of the responsible consultant spinal surgeon. Seventy-nine per cent of patients with a completed MSC required their C-spines to be cleared after critical care admission. When completed, the initial MSC allowed clearance of C-spine and immediate removal of hard collar in 67% of those patients. There were clearly documented instructions for C-spine care from a spinal consultant in 92% of patients with a completed MSC. Overall, an MSC was completed for only 39% of patients, despite 53% of patients having sustained a spinal fracture at some level (for example, lumbar, thoracic or cervical). The median time from critical care admission to MSC completion was 36 hours (range 3 hours to 12 days, mean 48 hours).

## Conclusion

The uptake of this checklist has not been optimal, but the MSC provides an excellent tool for clear documentation of C-spine status. During this initial trial phase, October 2010 to December 2011, the MSC has been consultant-only. Further action will involve rollingout the checklist to critical care trainee doctors to improve the rate of documentation of C-spine status and improve patient safety in this area of significant clinical risk [1].
